# Breastmilk influences development and composition of the oral microbiome

**DOI:** 10.1080/20002297.2022.2096287

**Published:** 2022-07-05

**Authors:** Catherine A. Butler, Geoffrey G. Adams, Jordan Blum, Samantha J. Byrne, Lauren Carpenter, Mark G. Gussy, Hanny Calache, Deanne V. Catmull, Eric C. Reynolds, Stuart G. Dashper

**Affiliations:** a Centre for Oral Health Research, Melbourne Dental School, University of Melbourne, Carlton, Vic, Australia; b Child and Community Wellbeing Unit, Melbourne School of Population & Global Health, University of Melbourne, Carlton, Vic, Australia; c Lincoln International Institute for Rural Health, College of Social Science, University of Lincoln, Lincoln, UK; d School of Health and Social Development, Deakin University, Burwood, Vic, Australia

**Keywords:** Oral cavity, breastfeeding, temporal development, early childhood, infant

## Abstract

**Background:**

Human microbiomes assemble in an ordered, reproducible manner yet there is limited information about early colonisation and development of bacterial communities that constitute the oral microbiome.

**Aim:**

The aim of this study was to determine the effect of exposure to breastmilk on assembly of the infant oral microbiome during the first 20 months of life.

**Methods:**

The oral microbiomes of 39 infants, 13 who were never breastfed and 26 who were breastfed for more than 10 months, from the longitudinal VicGeneration birth cohort study, were determined at four ages. In total, 519 bacterial taxa were identified and quantified in saliva by sequencing the V4 region of the bacterial *16S rRNA* genes.

**Results:**

There were significant differences in the development of the oral microbiomes of never breastfed and breastfed infants. Bacterial diversity was significantly higher in never breastfed infants at 2 months, due largely to an increased abundance of *Veillonella* and species from the *Bacteroidetes* phylum compared with breastfed infants.

**Conclusion:**

These differences likely reflect breastmilk playing a prebiotic role in selection of early-colonising, health-associated oral bacteria, such as the *Streptococcus mitis* group. The microbiomes of both groups became more heterogenous following the introduction of solid foods.

## Introduction

The human microbiome and human body constitute an integrated superorganism following coevolution, mutual adaptation and functional integration over hundreds of thousands of years [1]. The oral cavity is home to the second most diverse microbial community in the human body after the gut, with well over 700 bacterial taxa considered to be components of the human oral microbiome [2]. These bacteria colonise both the hard surfaces of teeth and soft tissues of the oral mucosa [3]. The oral microbiome is a complex microecosystem comprising a dynamic community of bacteria, the assembly of which is non-random and proposed to be dependent on early-life factors including host genetic factors, mode of birth delivery and nutritional source (formula or breastmilk) [4,5]. During and directly after birth, microbes colonise the oral cavity and the oral microbiome rapidly becomes more diverse and complex [6,7]. The first two years of life is a crucial period for development of the oral microbiome, with the core oral microbiome increasing from effectively zero at birth to over 32 species-level taxa at approximately two years of age [6]. Ecological succession is believed to occur with early bacterial colonisers of the oral cavity forming a scaffold for biofilm formation that develops into a complex microecosystem that persists over time. A biofilm is a community of microorganisms that adhere to each other and to surfaces; within the oral cavity there are several different kinds of surfaces including the tongue, buccal mucosa, keratinised gingiva, hard palate and teeth, all of which are colonised by bacterial communities and are continually shed into saliva, except for the non-shedding hard tooth surfaces, where semi-permanent biofilms form [8].

The influence of the infant’s diet on the relative composition and temporal assembly of the microbial communities that comprise the oral microbiome is currently poorly understood. A perturbed balance in the oral microbiome during infancy could disrupt the equilibrium of this oral ecosystem, carrying increased risk of oral and potentially systemic diseases [9,10]; this is certainly true of the gut microbiome [11,12]. Recent evidence suggests human genetics play a relatively minor role in the composition of the oral microbiome and that environmental influences appear to dominate the shaping of the oral microbiome [13,14]. The main dietary intake of the majority of Australian neonates and young infants is breastmilk [15], which is likely to play an important role in the temporal development of the oral microbiome, particularly as breastmilk contains its own bacterial community [16] and human milk oligosaccharides (HMOs). HMOs are one of the largest constituents of human breastmilk [17] functioning as prebiotics which allow the growth of beneficial bacterial taxa such as *Bifidobacterium* spp., while preventing the colonization of harmful pathogens, thereby promoting a health-associated microbiome [18–20]. The World Health Organization recommends exclusive breastfeeding during the first six months of life [21], and this window of breastmilk exposure is likely as important for oral bacterial community development, as it is for the gut microbiome [22]. As a child progresses from breastmilk to solid foods and sweetened drinks, this change in available nutrients for bacteria is pivotal in the emergence of oral disease [23], and the formation of a robust and resilient oral microbiome at an early age may promote oral health at later stages of life [24]. In fact, it has been reported that any exposure to breastmilk, in general, is protective against dental caries compared to children who never received breastmilk [25,26], but prolonged breastfeeding (> 12 months) is associated with dental caries [27].

We postulate that breastmilk constitutes an important factor in influencing the ordered temporal development of the infant oral microbiome. Therefore, the aim of this longitudinal study was to determine the significance of infant feeding on the temporal development of the oral microbiome, from approximately 2 months to 20 months of age.

## Methods

### Study population

Infants were selected, based on their exposure to mother’s own breastmilk, from the database of the longitudinal VicGeneration birth cohort study [28,29] for examination of their oral microbiome. Infant-mother pairs were recruited through Maternal and Child Health Centres in six local government areas in Victoria, Australia. Clinical oral examinations, questionnaires and collection of saliva were conducted by health professionals at 7 time points at approximately 2, 8, 13, 20, 39, 48 and 60 months of age. Unstimulated saliva, up to 5 mL, was collected from infants using a pipette and by passive drooling into a sterile tube. Data and saliva from the first four age time points were used in this study.

At each of the points, parents reported a) if their infant had **ever** received breastmilk; b) if their infant was currently receiving breastmilk (yes/no); and c) if no longer receiving breastmilk, the age (days/weeks/months) the infant stopped receiving breastmilk. Data were collated and used to allocate infants to one of two distinct groups; those who had **never** received breastmilk – never breastfed (NB), and those who had received breastmilk (whether exclusively or non-exclusively) for at least 10 months (B10), which captured the infants still being breastfed by the second time point. Infants in the B10 group were reported to have received breastmilk for a minimum of 40 weeks. Infants were excluded if more than one of their four time point samples were absent. Only thirteen infants met the criteria for inclusion into the NB group; each individual from the NB group was then age matched with two individuals from the B10 group across all four time points, using the Mahalanobis distance formula [30] to ensure maximal consistency of ages. This reduced the spread of ages of infants between the study groups [30]; the mean age of the infants (± standard deviation) at each of the four time points was 1.9 ± 0.8, 8.1 ± 1.0, 12.7 ± 1.0 and 20.1 ± 2.6 months. Four individuals in the NB group and three in the B10 group had only three saliva samples, the rest contained a complete set of four. Antibiotic usage information was not collected during the study. Three supplementary tables summarize the infant ages at the first four time points (Table S1), characteristics of the infants (Table S2) and characteristics of mothers (Table S3) at baseline.

### Ethics

Ethics approval to conduct the VicGeneration birth cohort study was provided by the University of Melbourne Human Research Ethics Committee (HREC 0722543 and HREC 1137124) and the study was approved by the Victorian Department of Education and Early Childhood Development (Ref: 2008/202). All research was performed in accordance with relevant guidelines and regulations, and informed consent was obtained from all participants or their parent or legal guardian. Consent for publication was provided as part of the Consent Form signed prior to participation in the study.

### 16S rRNA gene amplification and sequencing

Extraction of total DNA from saliva, amplification of the *16S rRNA* V4 region and sequencing using the Ion Torrent PGM were performed as previously reported [6]. The positive template control used was ZymoBIOMICS™ Microbial Community DNA Standard II (Log Distribution) (Zymo Research). The datasets generated and analysed during the current study are available from the NCBI Sequence Read Archive (SRA) repository using BioProject accession number PRJNA747639.

### Bioinformatic analyses

Fastq files were analysed using the DADA2 pipeline via the Nephele platform [31,32]. Within the DADA2 pipeline, the Human Oral Microbiome Database (HOMD) was used as the reference database for 16S rRNA identification of ASVs [33]. The following percentage identities were used to define levels of taxonomy: 98% for species, 95% for genus, 80% for phylum. Relative abundances of bacterial taxa were calculated, where the sequencing reads for one species was divided by the total number of reads from all species within one sample. Species were sorted according to average relative abundance across all samples, and the top 60 were checked to determine whether each species listed was part of a group of species that all shared the same sequence identity. Not all closely related bacteria can be differentiated by the V4 region alone of the *16S rRNA* gene; if there were two species with equal identity, they were both named using a slash call; if three or more had equal identity, they were listed as a group (Table S4). Generated files were used for bioinformatic and statistical analyses in RStudio, using the packages phyloseq, microbiome, ggpubr, ggplot2, vegan and RVAideMemoire. The *plot_richness* function from the phyloseq package was used to determine the species α-diversity (Shannon Diversity Index and Inverse Simpson Index) based on all species. β-diversity was calculated using Principal Coordinate Analysis (PCoA) at the genus level (*ordinate* function from phyloseq package using the weighted UniFrac distance metric option), restricted to species with a minimum relative abundance of 0.001 and minimum prevalence of 10%. For comparisons of relative abundance results and α-diversity results between the NB and B10 groups the Mann–Whitney test was used. For β-diversity, overall and pairwise comparisons were performed using permutational MANOVAs (functions *adonis, pairwise.perm.manova* and *betadisper*), all in RStudio. The Benjamini-Hochberg False Discovery Rate correction was used to adjust p-values for multiple comparisons.

## Results

### Relative abundances of bacterial taxa in infant saliva

Sequencing of the 149 saliva samples generated a total of approximately 7.69 million 16S DNA V4 sequencing reads, with an average of 51,600 reads per sample. Ten phyla accounted for all bacteria identified in this study. The five most abundant phyla (*Firmicutes, Proteobacteria, Bacteroidetes, Actinobacteria* and *Fusobacteria*) accounted for 99.5% of the total abundance. The most abundant phylum observed across all samples and time points was *Firmicutes*, with a mean relative abundance of 0.77 ± 0.15 (± SD). The average relative abundance of *Firmicutes* was highest in infants ~2 months of age in both study groups, which decreased at each subsequent time point, and this was the only phylum to exhibit this trend. The average relative abundance of each phylum followed similar patterns of change across the time points for both NB and B10 groups, apart from the *Bacteroidetes*, which had a five-fold higher average relative abundance in NB infants than B10 infants at ~2 months of age. The B10 infants attained a similar average relative abundance of *Bacteroidetes* by the second time point of ~8 months of age. No major differences were observed for bacteria belonging to other phyla.

**Figure 1.**
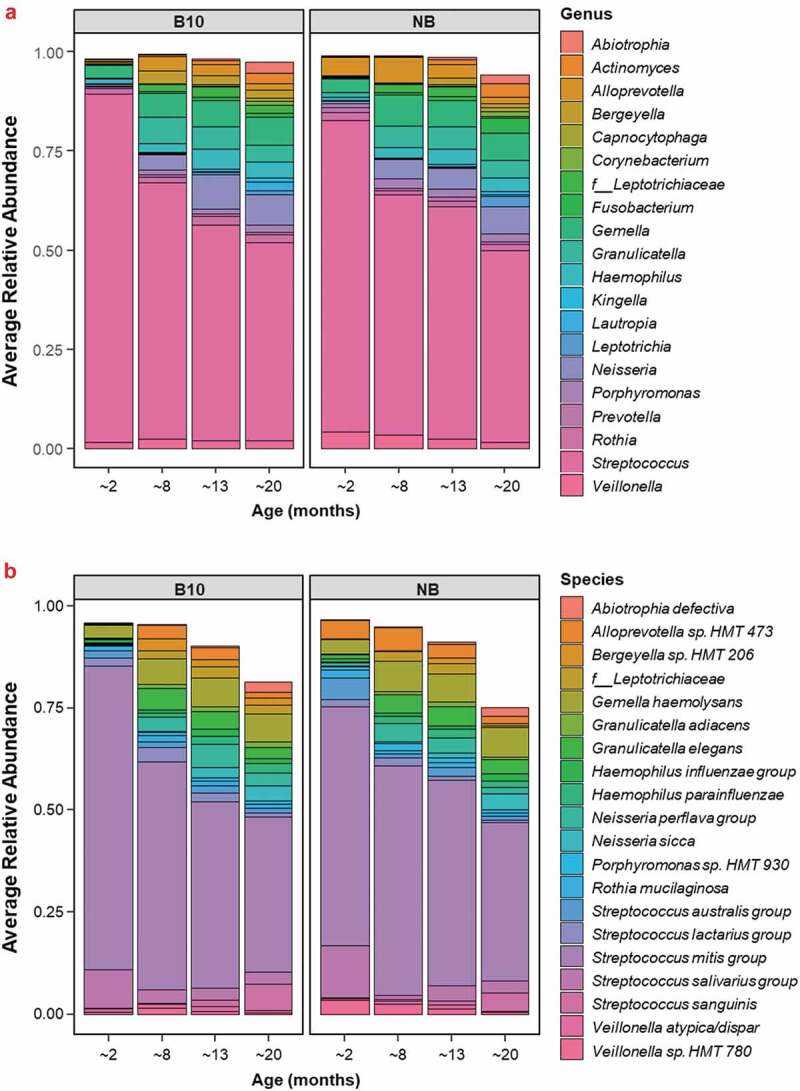
The 20 most abundant bacterial (**a**) genera and (**b**) species detected in infant saliva at 1.9 months of age according to the study group and their change in abundance over time. The relative abundances of the 20 most abundant bacterial genera/species were calculated as a proportion of the total salivary bacterial species within each sample, as determined by Ion Torrent PGM™ sequencing of the *16S rRNA* gene V4 region. The relative abundances of the top 20 bacterial genera/species were averaged across infants within each study group to give Average Relative Abundance, at each of the four sampling ages. ‘*f*__*Leptotrichiaceae*’ is named as such as this particular taxon was only identifiable to the family level.

At the genus level, *Streptococcus* dominated the oral microbiome across all samples, decreasing with age until it comprised ~50% of the salivary bacteria by ~20 months of age (Figure 1(a)). The bacterial genus *Alloprevotella* was significantly more abundant in NB infants at ~2 months of age compared with B10 infants at this age (p = 0.027). *Alloprevotella* belongs to the *Bacteroidetes* phylum, consistent with the increase in *Bacteroidetes* observed in the NB group at this age.

**Figure 2.**
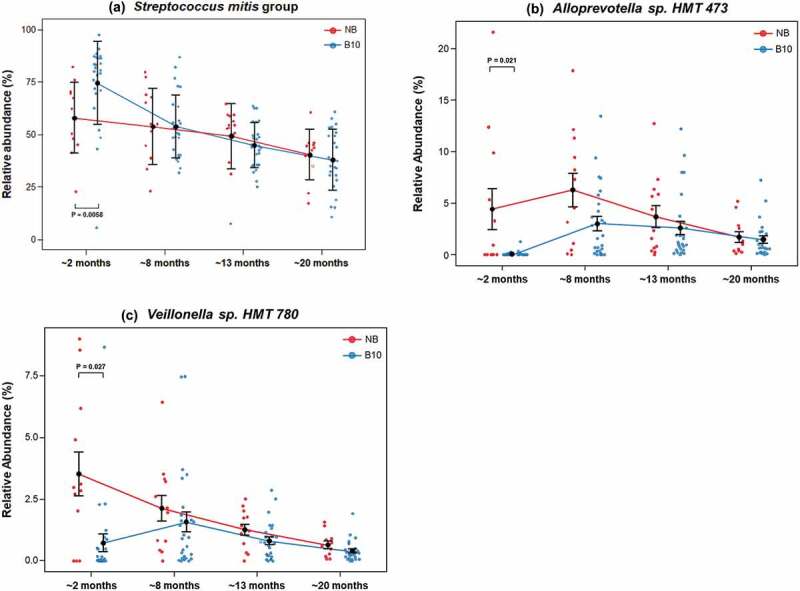
Bacterial species with significantly different average relative abundances between B10 and NB infants. Average relative abundances of **a**. *Streptococcus mitis* group, **b**. *Alloprevotella* sp. HMT 473 and **c**. *Veillonella* sp. HMT 780, across the four sampling ages. P-values are shown for statistically significant differences in average relative abundance between study groups. Solid blue lines represent the B10 group, solid red lines represent the NB group. Means and standard errors are shown in black.

Of the 519 bacterial taxa identified across all samples, the 20 most abundant species accounted for an average of 89% of the total bacteria across all sampling ages (Figure 1(b)). The three most abundant bacterial taxa, the *Streptococcus mitis* group, *Gemella haemolysans* group and *Streptococcus salivarius* group, comprised an average of 63% of the total bacteria found in the saliva of infants across all sampling ages, and an average of 81% of the total bacteria in infants ~2 months of age (Figure 1(b), Table 1). At all sampling ages, the *S. mitis* group was the most abundant bacterial taxon in infant saliva (Figure 1(b), Table 1). The average relative abundance of the *S. mitis* group was significantly higher in the B10 infants compared with the NB infants at ~2 months of age (p = 0.006; Figure 2(a)). After this age, the average relative abundance of the *S. mitis* group progressively decreased from ~69% at ~2 months of age to ~39% by ~20 months of age across both study groups. *Alloprevotella* sp. HMT 473 was significantly higher in average relative abundance in NB infants ~2 months of age (Figure 2(b)), compared with B10 infants (p = 0.021). Over time, there was less variation between samples in each infant group for this bacterium. The average relative abundance of *Veillonella* sp. HMT 780 was significantly higher in NB infants compared with B10 infants at ~2 months of age (p = 0.027; Figure 2(c)), suggesting a preference for this bacterium to colonise the oral cavity of never breastfed infants during early stages of life. Although not statistically significant, there was a trend for the average relative abundance of *Porphyromonas* sp. HMT 930 to be higher in NB infants across all sampling ages (Table 1), and for *Lautropia mirabilis* to be found in higher relative abundance in B10 infants across all sampling ages (Table 1).

**Table 1.** Temporal development of the core oral microbiome. Prevalence and average relative abundance of bacterial species per study group and time point. Prevalence values represent the percentage of infants within each study group with detectable levels of each taxa at the four mean ages tested. Shaded values indicate bacterial taxa considered as core (> 81% prevalence). Taxa are arranged from early to late colonisers, and the total number of core taxa at each time point is shown. If two species could not be differentiated using the V4 region, both are shown as a slash call; if three or more species could not be differentiated, they are named as groups (Table S4). Bacterial species were not included that had a prevalence greater than 81% at only a single age and study group, ensuring exclusion of transient bacterial taxa that may otherwise have been considered as core.

	~2 months				~8 months				~13 months				~20 months			
	Prevalence		Relative Abundance		Prevalence		Relative Abundance		Prevalence		Relative Abundance		Prevalence		Relative Abundance	
Species	NB	B10	NB	B10	NB	B10	NB	B10	NB	B10	NB	B10	NB	B10	NB	B10
*Streptococcus mitis group*	100%	100%	0.5809	0.7459	100%	100%	0.5385	0.5390	100%	100%	0.4933	0.4494	100%	100%	0.4021	0.3795
*Gemella haemolysans group*	100%	100%	0.0393	0.0373	100%	100%	0.0781	0.0679	100%	100%	0.0646	0.0710	100%	100%	0.0749	0.0697
*Streptococcus salivarius group*	100%	96%	0.1332	0.0810	100%	100%	0.0101	0.0354	100%	100%	0.0427	0.0303	100%	100%	0.0337	0.0275
*Rothia mucilaginosa*	100%	92%	0.0165	0.0125	100%	100%	0.0087	0.0145	100%	100%	0.0137	0.0151	100%	100%	0.0079	0.0105
*Alloprevotella sp. HMT 473*	100%	60%	0.0439	0.0010	100%	100%	0.0627	0.0305	100%	100%	0.0380	0.0260	100%	100%	0.0175	0.0151
*Streptococcus australis group*	92%	76%	0.0473	0.0154	100%	96%	0.0114	0.0202	100%	100%	0.0232	0.0183	100%	100%	0.0128	0.0127
*Veillonella atypica/dispar*	83%	88%	0.0074	0.0088	100%	100%	0.0100	0.0113	92%	85%	0.0120	0.0108	91%	88%	0.0035	0.0073
*Veillonella sp. HMT 780*	83%	76%	0.0349	0.0073	92%	100%	0.0221	0.0161	92%	96%	0.0128	0.0082	100%	100%	0.0066	0.0040
*Granulicatella elegans*	83%	76%	0.0088	0.0020	100%	100%	0.0453	0.0547	100%	100%	0.0421	0.0465	100%	100%	0.0353	0.0264
*Bergeyella sp. HMT 931*	83%	72%	0.0004	0.0014	92%	88%	0.0008	0.0007	92%	85%	0.0011	0.0009	91%	75%	0.0006	0.0003
*Granulicatella adiacens*	58%	40%	0.0026	0.0015	100%	100%	0.0104	0.0103	100%	100%	0.0101	0.0126	100%	100%	0.0082	0.0133
*Neisseria perflava group*	67%	68%	0.0110	0.0027	100%	96%	0.0480	0.0326	100%	100%	0.0392	0.0574	100%	100%	0.0199	0.0340
*Streptococcus lactarius group*	58%	64%	0.0193	0.0210	100%	100%	0.0204	0.0373	100%	96%	0.0091	0.0210	82%	75%	0.0045	0.0093
*Haemophilus influenzae group*	50%	68%	0.0056	0.0087	92%	100%	0.0091	0.0095	92%	100%	0.0116	0.0196	100%	96%	0.0205	0.0133
*Porphyromonas pasteri/sp. HMT 278*	67%	28%	0.0011	0.0002	100%	88%	0.0081	0.0044	100%	96%	0.0068	0.0057	100%	100%	0.0096	0.0093
*Porphyromonas sp. HMT 930*	67%	48%	0.0083	0.0007	92%	92%	0.0174	0.0075	92%	92%	0.0102	0.0060	100%	100%	0.0088	0.0064
*Actinomyces sp. HMT 180/odontolyticus*	50%	52%	0.0022	0.0020	92%	92%	0.0026	0.0036	92%	92%	0.0031	0.0035	100%	96%	0.0018	0.0034
*Haemophilus parainfluenzae*	33%	60%	0.0004	0.0024	92%	85%	0.0185	0.0128	100%	100%	0.0155	0.0203	100%	100%	0.0163	0.0202
*Prevotella melaninogenica*	50%	24%	0.0054	0.0008	83%	85%	0.0022	0.0022	100%	85%	0.0031	0.0023	100%	92%	0.0016	0.0036
*Campylobacter concisus*	33%	28%	0.0007	0.0004	83%	81%	0.0003	0.0009	92%	85%	0.0013	0.0007	91%	100%	0.0015	0.0008
*Fusobacterium periodonticum*	17%	16%	0.0000	0.0002	92%	65%	0.0038	0.0021	100%	85%	0.0045	0.0050	100%	88%	0.0123	0.0045
*Prevotella nanceiensis*	33%	20%	0.0007	0.0001	83%	69%	0.0025	0.0012	100%	96%	0.0010	0.0018	82%	100%	0.0008	0.0019
*Bergeyella sp. HMT 322*	25%	16%	0.0003	0.0001	83%	65%	0.0011	0.0005	92%	88%	0.0010	0.0014	100%	100%	0.0022	0.0027
*Bergeyella sp. HMT 206*	33%	44%	0.0000	0.0039	83%	62%	0.0010	0.0315	92%	81%	0.0157	0.0206	82%	79%	0.0077	0.0176
*Streptococcus sanguinis*	0%	8%	0.0000	0.0013	50%	54%	0.0040	0.0033	100%	100%	0.0104	0.0159	100%	100%	0.0423	0.0634
*Neisseria sicca*	0%	20%	0.0000	0.0001	33%	42%	0.0051	0.0041	100%	100%	0.0131	0.0249	100%	100%	0.0386	0.0399
*Abiotrophia defectiva*	8%	24%	0.0000	0.0002	58%	54%	0.0007	0.0009	92%	96%	0.0056	0.0063	100%	100%	0.0220	0.0271
*Rothia aeria*	0%	8%	0.0000	0.0004	17%	19%	0.0006	0.0004	85%	81%	0.0026	0.0054	91%	96%	0.0040	0.0095
*Lautropia mirabilis*	8%	16%	0.0000	0.0002	33%	31%	0.0007	0.0011	77%	96%	0.0016	0.0047	100%	100%	0.0073	0.0231
*Actinomyces naeslundii group*	0%	8%	0.0000	0.0006	25%	31%	0.0007	0.0014	77%	96%	0.0063	0.0047	100%	100%	0.0190	0.0208
*Capnocytophaga sputigena*	0%	4%	0.0000	0.0000	33%	35%	0.0005	0.0003	77%	88%	0.0010	0.0010	100%	100%	0.0032	0.0049
*Lachnoanaerobaculum umeaense*	33%	8%	0.0005	0.0000	75%	65%	0.0007	0.0005	77%	88%	0.0007	0.0007	100%	92%	0.0013	0.0018
*Corynebacterium durum*	0%	12%	0.0000	0.0001	8%	8%	0.0000	0.0005	62%	73%	0.0009	0.0015	100%	96%	0.0023	0.0054
*Fusobacterium nucleatum group*	17%	16%	0.0017	0.0004	17%	8%	0.0003	0.0010	62%	54%	0.0011	0.0002	100%	96%	0.0081	0.0044
*Kingella/Neisseria denitrificans/elongata*	0%	4%	0.0000	0.0000	17%	27%	0.0010	0.0018	62%	77%	0.0020	0.0045	100%	92%	0.0035	0.0072
*Capnocytophaga leadbetteri*	0%	12%	0.0000	0.0000	25%	31%	0.0002	0.0001	62%	73%	0.0008	0.0003	100%	92%	0.0016	0.0012
*Pseudopropionibacterium sp. HMT 194*	0%	8%	0.0000	0.0001	17%	4%	0.0003	0.0002	31%	23%	0.0003	0.0004	100%	92%	0.0022	0.0026
*Cardiobacterium hominis*	0%	8%	0.0000	0.0000	8%	8%	0.0002	0.0001	69%	58%	0.0004	0.0005	91%	88%	0.0018	0.0022
*Streptococcus pneumoniae*	17%	24%	0.0000	0.0001	33%	42%	0.0001	0.0005	31%	73%	0.0009	0.0008	82%	83%	0.0019	0.0009
**Total number of core microbiome taxa**	**10**	**5**	**0**	**0**	**24**	**20**	**0**	**0**	**28**	**32**	**0**	**0**	**39**	**36**	**0**	**0**

### Core oral taxa

To aid in our understanding of how the oral microbiome develops over time, we defined a ‘core’ oral microbiome as containing bacterial taxa that had a prevalence greater than 81% at one or more ages, for the two study groups (Table 1). This percentage was chosen so that taxa from the limiting study group were considered ‘core’ if they were absent from a maximum of 2 infants (n = 11 for NB infants at 19.8 months). At ~2 months of age, an average of just over seven bacterial taxa were considered as core across the study groups, and the number of core bacteria was seen to increase with age (Table 1). At ~2 months of age, more bacterial taxa were considered as core in the NB infants (10 taxa) compared to the B10 infants (five taxa), suggesting a correlation between infants that had never received breastmilk and a propensity for earlier colonisation by core oral taxa. The average relative abundance of the majority of the 10 core taxa of the NB group was greater in the NB group than the B10 group at ~2 months. Regardless of study group, the *S. mitis* group, *G. haemolysans* group, *Streptococcus salivarius* group, *Rothia mucilaginosa* and *V. atypica/dispar* were considered as core bacterial taxa from the first sampling age and remained as core at all subsequent sampling ages (Table 1).

### Alpha and βeta diversity

**Figure 3.**
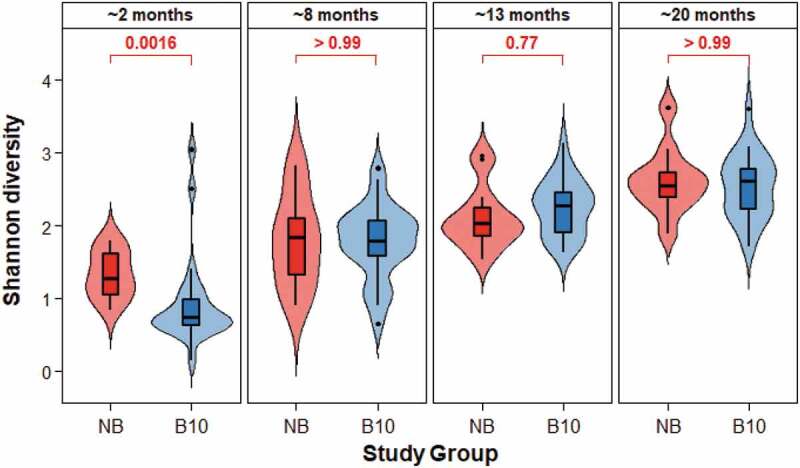
Violin and boxplot of the bacterial α-diversity within infant saliva according to age and study group. Bacterial α-diversity was measured according to the Shannon Diversity Index. Data from the B10 group is blue and from the NB group is red. The violin plot shows the kernel probability density of the data at different values. The boxplot represents the same data in quartiles, with the horizontal line in the boxplot representing the median, and the ‘box’ representing 50% of the data. The upper and lower whiskers of the boxplot represent values of 1.5 multiplied by the IQR. Mean comparison p-values are given between the different study groups.

At the species level, α-diversity increased with age for infants belonging to both study groups (Figure 3). At ~2 months of age, NB infants had significantly greater bacterial α-diversity than infants in the B10 study group (Figure 3, Shannon Diversity Index, p = 0.0016; Figure S1, Inverse Simpson Diversity Index, p = 0.0058).

**Figure 4.**
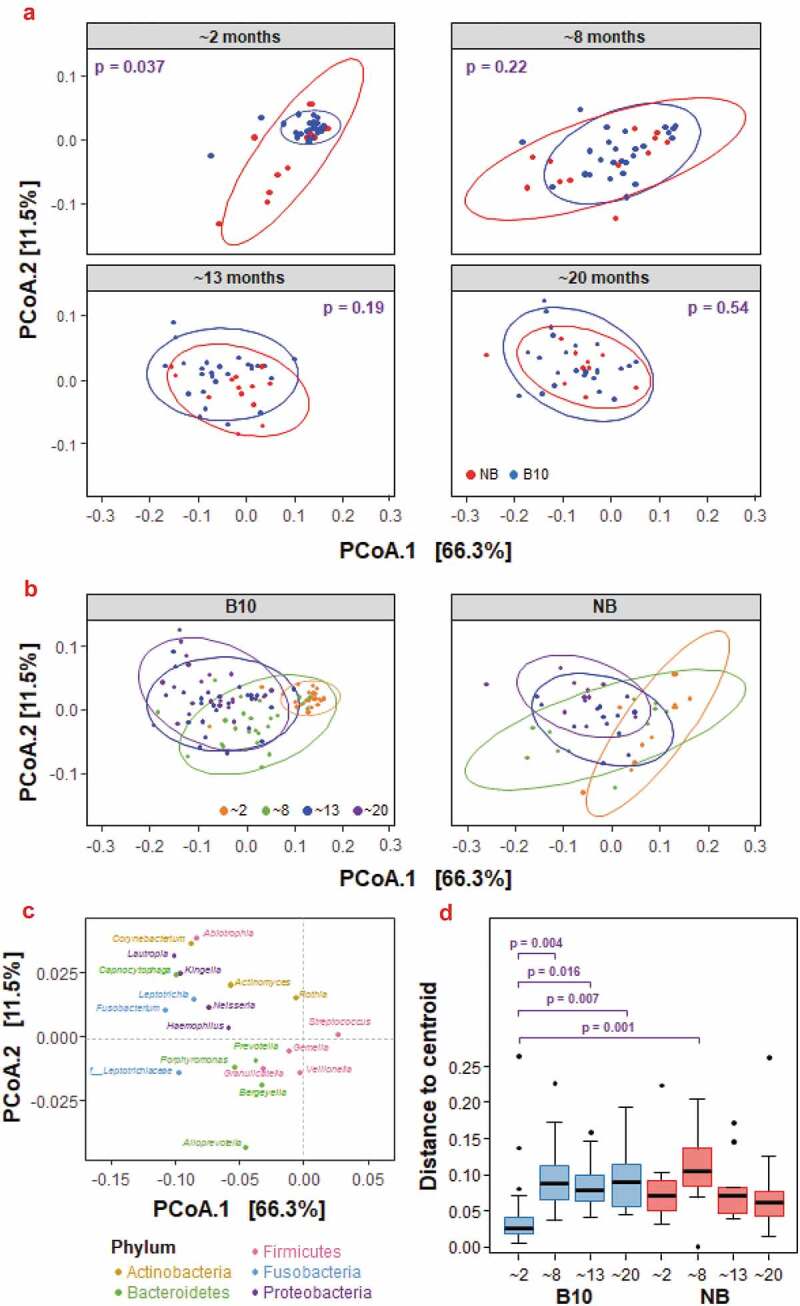
Principal coordinate analysis (PCoA) of the β-diversity of abundant bacterial genera from each infant, across study groups and ages. **(a)** Data are shown for each study group (B10 = blue; NB = red) at each of the four time points, using weighted UniFrac distance metrics. **(b)** Data are shown at each of the four time points for the two study groups, using weighted UniFrac distance metrics. 95% confidence ellipses are also shown for each study group. Statistical significance was determined via pairwise comparisons using permutational MANOVAs. **(c)** The species loadings are shown for the top 20 genera. The genus *Streptococcus* was positively correlated with the PCoA.1 dimension (r = 0.96) and the genera *Neisseria* and *Haemophilus* were negatively correlated with the PCoA.1 dimension (r = – 0.77 and r = – 0.62, respectively). The genus *Alloprevotella* was negatively correlated with the PCoA.2 dimension (r = – 0.74). **(d)** Differences in group homogeneities are shown each of the four time points for the two study groups ((B10 = blue; NB = red). P-values are shown for statistically significant differences between study groups.

To elucidate differences in bacterial composition between saliva samples from breastfed and never breastfed infants, PCoA was performed for abundant bacterial genera (Figure 4). Genera were included if their associated species had a relative abundance greater than 0.001 in at least 10% of all samples. A total of 71 species satisfied this criterion, which translated to 40 genera being used for PCoA. At ~2 months of age, the bacterial β-diversity of NB infants was significantly greater than B10 infants (Figure 4a; p=0.037), with the B10 infants showing significantly greater homogeneity at ~2 months of age than at any other time point (Figure 4b, Figure 4d), as their saliva was dominated by the *S. mitis* group. As the infants grew, their salivary microbiome became more diverse, reflecting more heterogeneity between infants (Table 1). The B10 infants at ~8 months had significantly greater β-diversity when compared to B10 infants at ~2 months (p = 0.0028), and there were further shifts in composition at ~13 and ~ 20 months (~8 months vs ~13 months – p = 0.0043; ~13 months vs ~20 months – p = 0.0028). Infants belonging to the NB study group also had microbial communities that grew more diverse over time, with the bacterial composition in NB infants at ~2 months of age significantly different from the composition at all later time points (~8 months – p = 0.025; ~13 months – p = 0.0043; ~20 months – p = 0.0028). Furthermore, as the infants aged, the salivary composition of the two groups became more similar, the 95% confidence ellipses overlapped more, and no significant differences were found in β-diversity between the two groups at ~ 8 months (p = 0.22), ~13 months (p = 0.19) and ~20 months (p = 0.54).

## Discussion

There were significant differences in the temporal development of the oral microbiomes of never breastfed and breastfed infants, despite the overall trend being similar. Whilst the phylum *Firmicutes* dominated in all samples at all time points, the never breastfed infants had a greater abundance of bacteria belonging to the phylum *Bacteroidetes*, compared with breastfed infants at ~2 months of age. A similar difference in the relative abundance of the phylum *Bacteroidetes* was previously observed in a comparison of the oral microbiome in formula-fed and breastfed infants in a cross-sectional study of infants under 2 months of age [34]. In the current study, two species from phylum *Bacteroidetes* (*Alloprevotella* sp. HMT 473 and *Porphyromonas* sp. HMT 930) were found in higher abundance in never breastfed infants (Table 1).

Six species (*Alloprevotella* sp. HMT 473, *Streptococcus australis* group, *V. atypica/dispar, Veillonella* sp. HMT 780, *Granulicatella elegans* and *Bergeyella* sp. HMT 93) were found to colonise the oral cavity of never breastfed infants earlier than breastfed infants (Table 1). This indicated a shift in the bacterial colonisation of the oral cavity and bacterial community development in never breastfed infants compared with infants who received breastmilk. This was also reflected in significantly greater oral bacterial α- and β-diversity in never breastfed infants compared with infants that received breastmilk during the early stages of life (Figure 3 and 4) [34,35]. Although the most abundant taxa in all samples (Figure 1), there was significantly more *S. mitis* group bacteria in the breastfed infants at 2 months of age (Figure 2A). The homogeneity of the early oral microbiome seen in the breastfed infants was most likely due to the bioactive components and commensal bacteria found in human breastmilk, that are not found in formula, guiding microbiome development. Breastmilk contains antimicrobial peptides, immunoglobulins, HMOs and commensal bacteria including *Streptococcus*; all of which boost the infant immune system and provide defence against pathogens [36]. Secretory IgA, the primary protective antibody in breastmilk, prevents pathogen adherence to epithelial cell surfaces and neutralises toxins [37]. HMOs are a group of complex carbohydrates that are highly abundant in human milk (10–15 g/L) but are not digested by the infant and the majority reach the colon. Their impact on the oral microbiome has been poorly studied but the ability of early colonisers such as *Streptococcus* spp., which are found in breastmilk, to catabolise HMOs and the effects of the end products of this catabolism, could enable them to dominate the early infant oral microbiome of breastfed infants and provide the foundation for the guided development of the oral microbiome. A recent preliminary study has shown that the growth of both *S. mitis* and *Streptococcus oralis* was promoted by the HMO 2’-fucosyllactose, which was attributed to the presence of the enzyme fucosidase in these species [38]. This is consistent with the ability of early colonising oral streptococci to grow on highly glycosylated salivary mucins and the relatively high abundance of genes encoding glycosidases, sugar transporters and glycan binding proteins in their genomes. *S. mitis* has been shown to inhibit the colonization of other bacterial species, including potential pathogens [39]. HMOs have also been shown to directly inhibit the growth of pathogenic bacteria including *Streptococcus agalactiae* (Group B *Streptococcus), Staphylococcus aureus* and *Acinetobacter baumannii* and to disrupt their biofilm formation [40–42]. The microbiome of breastmilk can also be influenced by retrograde backflow of milk where bacteria from the infant’s mouth is transferred into the mother’s mammary gland [36]. Collectively, these mechanisms are likely to help shape the early infant oral microbiome and are at least in part responsible for the significant differences seen between breastfed and never breastfed groups in this study (Figures 1 to 4, Table 1).

The species *Veillonella* sp. HMT 780 was significantly higher in relative abundance in never breastfed infants at ~2 months of age compared with B10 infants (Figure 1, Figure 2C). This finding was consistent with previous literature that found *Veillonella* sp. HMT 780 associated with exclusively formula-fed infants at three months of age [5]. Various studies have reported that *Veillonella* sp. HMT 780 was significantly more abundant in children affected by severe early childhood caries, compared with children who were caries-free [43,44]. *Veillonella* spp. are not cariogenic, however they are dependent on the lactic acid produced by cariogenic bacteria as an energy source [45]. The presence of this bacterial genus in elevated proportions is likely indicative of a diet with considerable free sugars that favours an acidogenic bacterial biofilm. A significantly higher relative abundance of salivary *Veillonella* sp. HMT 780 in the never breastfed infants suggests a lower salivary pH, which could potentially exclude commensal neutrophilic bacteria from colonising the oral cavity, contributing to disease-associated bacterial community development.

Alpha and beta diversity analyses together indicated that the temporal changes occurring in the two groups were similar, but they started from different points as measured at ~2 months. This difference was largely due to the *S. mitis* group accounting for 75% of the microbiota in infants exposed to breastmilk. By 8 months of age all infants were eating solid food (Table S2), and any early benefit from breastmilk was no longer detectable. As more species became core (Table 1), there was an increasing heterogeneity of the microbiome with less emphasis on *Streptococcus* spp. (Figure 4).

Limitations of this study include the small sample size which was dictated by the small number of participants in the longitudinal VicGeneration birth cohort study that had never been exposed to mother’s own breastmilk. The types of infant formula consumed by the NB infants and used to supplement the B10 infants’ diet were not recorded during this study, so it is not possible to determine whether particular formulations influenced the composition of the microbiome. Antibiotic usage data were also not collected, potentially leading to altered bacterial composition. It is clear however, that exposure to breastmilk influenced the composition of the oral microbiome, particularly at ~2 months of age when 66% of the breastfed subjects were exclusively breastfed.

In conclusion, exposure to breastmilk has an observable influence over the development of the infant oral microbiome. The incorrect order of oral microbial colonisation of infants not only has ramifications for development of future oral disease such as caries but may also interfere with ordered gut colonisation. Recent parallel tracking of salivary and gut microbiota profiles of early life microbial communities in healthy infants indicated that oral *Streptococcus* and *Veillonella* are involved in gut microbiota development as seeding species [46]. More research is required into understanding how differences in temporal development of the infant oral microbiome can result in oral disease such as dental caries. This could potentially identify microbial biomarkers for prediction of children with an increased risk of developing dental caries.

## Supplementary Material

Supplemental Material

## Data Availability

Datasets generated and analysed during the current study are available from the NCBI Sequence Read Archive (SRA) repository using BioProject accession number PRJNA747639. https://www.ncbi.nlm.nih.gov/bioproject/?term=PRJNA747639
