# Supplementary material for: Breastmilk influences development and composition of the oral microbiome
Source: J Oral Microbiol. 2022 Jul 5;14(1):2096287. doi: 10.1080/20002297.2022.2096287 (PMC9272919; doi:10.1080/20002297.2022.2096287)
Supplement: Supplemental Material [file ZJOM_A_2096287_SM8404.docx]

**Supplementary Tables and Figures**

**Table S1.** Infant Age at Study Visits (months)

|  | B10 | NB |
| --- | --- | --- |
| Time point 1 |  |  |
| N | 26 | 13 |
| Mean ± SD | 1.96 ± 0.80 | 1.86 ± 0.68 |
| Range | 0.95 - 3.98 | 0.99 - 2.99 |
| Time point 2 |  |  |
| N | 26 | 12 |
| Mean ± SD | 8.04 ± 0.90 | 8.19 ± 1.16 |
| Range | 6.57 - 9.83 | 6.77 - 9.80 |
| Time point 3 |  |  |
| N | 26 | 13 |
| Mean ± SD | 12.77 ± 0.89 | 12.48 ± 1.28 |
| Range | 11.37 - 14.40 | 10.26 - 15.45 |
| Time point 4 |  |  |
| N | 26 | 12 |
| Mean ± SD | 20.04 ± 2.40 | 20.33 ± 3.00 |
| Range | 17.29 - 25.84 | 17.13 - 26.04 |

**Table S2.** Characteristics of infants

| Attribute | B10 | NB |
| --- | --- | --- |
| Sex of Infant |  |  |
| Female | 12 (48%) | 9 (75%) |
| Male | 14 (56%) | 4 (33%) |
| Infant Birth Weight (kg) | 3.5 ± 0.6 | 3.5 ± 0.4 |
| Infant Birth Length (cm) | 50.4 ± 2.2 | 50.1 ± 1.5 |
| Length of Pregnancy (wks) | 39.6 ± 1.2 | 39.6 ± 1.7 |
| Infant Order |  |  |
| 1st child | 14 (56%) | 2 (17%) |
| 2nd child | 4 (16%) | 6 (50%) |
| 3rd child | 6 (24%) | 4 (33%) |
| 4th child or more | 2 (8%) | 1 (8%) |
| Duration of breastfeeding (wks) |  |  |
| N | 26 | 13 |
| Mean ± SD | 64.1 ± 16.2 | 0.0 ± 0.0 |
| Range | 43.5 - 107.4 | 0.0 - 0.0 |
| Age formula started (wks) |  |  |
| N | 20 | 13 |
| Mean ± SD | 16.3 ± 15.5 | 0.0 ± 0.0 |
| Range | 0.4 - 44.0 | 0.0 - 0.0 |
| Age other milk started (wks) |  |  |
| N | 26 | 11 |
| Mean ± SD | 44.2 ± 9.4 | 48.9 ± 11.1 |
| Range | 26.0 - 61.0 | 35.0 - 78.0 |
| Age solid foods started (wks) |  |  |
| N | 25 | 13 |
| Mean ± SD | 23.9 ± 3.4 | 20.1 ± 4.8 |
| Range | 17.0 - 30.0 | 13.0 - 26.0 |

**Table S3.** Characteristics of mothers

| Attribute | **B10** | **NB** |
| --- | --- | --- |
| Age of Parent (Mean ± SD) | 31.9 ± 4.3 | 28.5 ± 7.0 |
| Parent Region of Birth |  |  |
| Australia | 19 (76%) | 12 (100%) |
| North Africa & Middle East | 1 (4%) | 0 (0%) |
| North-East Asia | 1 (4%) | 0 (0%) |
| Oceania & Antarctica | 1 (4%) | 0 (0%) |
| South-East Asia | 3 (12%) | 1 (8%) |
| No Country Specified | 1 (4%) | 0 (0%) |
| Language at home |  |  |
| English | 22 (88%) | 11 (92%) |
| Arabic | 1 (4%) | 0 (0%) |
| Macedonian | 1 (4%) | 0 (0%) |
| Mandarin | 1 (4%) | 0 (0%) |
| Turkish | 0 (0%) | 1 (8%) |
| Vietnamese | 0 (0%) | 1 (8%) |
| No Language Specified | 1 (4%) | 0 (0%) |
| Level of Schooling |  |  |
| Year 12 | 24 (96%) | 8 (67%) |
| Year 11 | 0 (0%) | 4 (33%) |
| Year 10 or less | 2 (8%) | 1 (8%) |
| Post School Qualification |  |  |
| Advanced Diploma | 1 (4%) | 2 (17%) |
| Certificate | 4 (16%) | 4 (33%) |
| Degree (bachelor level) | 12 (48%) | 0 (0%) |
| Graduate Diploma/Certificate | 0 (0%) | 2 (17%) |
| Postgraduate Degree | 6 (24%) | 0 (0%) |
| Technical Apprenticeship | 1 (4%) | 0 (0%) |
| None | 2 (8%) | 5 (42%) |
| Health Care Card |  |  |
| Yes | 6 (24%) | 8 (67%) |
| No | 20 (80%) | 5 (42%) |
| Rurality |  |  |
| Metro | 16 (64%) | 8 (67%) |
| Regional | 1 (4%) | 1 (8%) |
| Rural | 9 (36%) | 4 (33%) |
| SEIFA IRSD (deciles) |  |  |
| 1 - 2 | 15 (60%) | 7 (58%) |
| 3 - 4 | 1 (4%) | 3 (25%) |
| 5 - 6 | 5 (20%) | 2 (17%) |
| 7 - 8 | 4 (16%) | 1 (8%) |
| 9 - 10 | 1 (4%) | 0 (0%) |

**Table S4.** List of bacterial groups with the slash call bacterial species comprising each group, from the top 20 most abundant bacterial taxa.

| Name | Slash Call |
| --- | --- |
| Streptococcus mitis group | *Streptococcus mitis/oralis/infantis/*sp. HMT 061*/*sp. HMT 064*/*sp. HMT 423 |
| Gemella haemolysans group | *Gemella haemolysans/sanguinis/morbillorum* |
| Streptococcus salivarius group | *Streptococcus salivarius/thermophilus/vestibularis* |
| Neisseria perflava group | *Neisseria perflava/subflava/cinerea/flavescens* |
| Streptococcus australis group | *Streptococcus australis/parasanguinis/*sp. HMT 066*/*sp. HMT 057 |
| Streptococcus lactarius group | *Streptococcus lactarius/peroris/*sp. HMT 074 |
| Neisseria sicca group | *Neisseria sicca/flava/macacae/mucosa* |
| Haemophilus influenzae group | *Haemophilus influenzae/haemolyticus/*sp. HMT 036*/*sp. HMT 259*/*sp. HMT 908 |
| Actinomyces naeslundii group | *Actinomyces naeslundii/oris/* sp. HMT 169*/*sp. HMT 171*/*sp. HMT 175 |
| Fusobacterium nucleatum group | *Fusobacterium nucleatum subsp. polymorphum/*sp. HMT 203 |


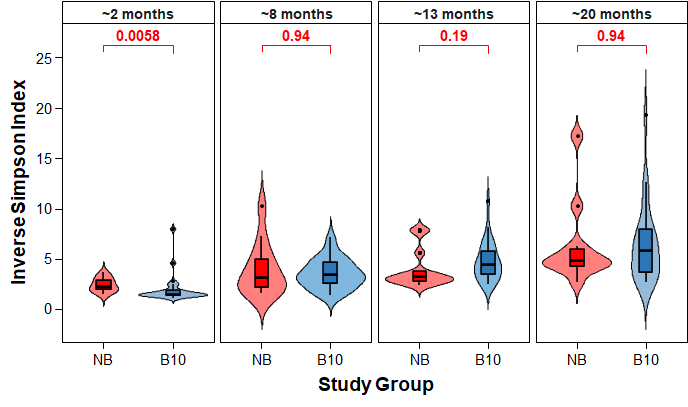


**Figure S1.** Violin and boxplot of the bacterial α-diversity within infant saliva according to mean age and study group. Bacterial α-diversity is measured according to the Inverse Simpson Index. Data from the B10 group are blue, and from the NB group are red. The violin plot shows the kernel probability density of the data at different values. The boxplot represents the same data in quartiles, with the horizontal line in the boxplot representing the median, and the ‘box’ representing 50% of the data. The upper and lower whiskers of the boxplot represent values of 1.5 multiplied by the IQR. Mean comparison p-values are given between study groups.
